# Middle lobe suffering due to malposition and 180° tilt of the 2 remaining lobes after right upper lobectomy

**DOI:** 10.1093/icvts/ivad038

**Published:** 2023-02-22

**Authors:** Aurelie Janet-Vendroux, Charbel Al Zreibi, Guillaume Reverdito, Alex Arame, Alain Badia, Hicham Masmoudi, Houssem Messaoudi, Francoise Le Pimpec-Barthes

**Affiliations:** Department of Thoracic Surgery and Lung Transplantation, Hôpital Européen Georges-Pompidou, Assistance Publique—Hôpitaux de Paris, Université Paris Cité, Paris, France; Department of Thoracic Surgery and Lung Transplantation, Hôpital Européen Georges-Pompidou, Assistance Publique—Hôpitaux de Paris, Université Paris Cité, Paris, France; Department of Radiology, Hôpital Européen Georges-Pompidou, Assistance Publique-Hôpitaux de Paris, Université Paris Cité, Paris, France; Department of Thoracic Surgery and Lung Transplantation, Hôpital Européen Georges-Pompidou, Assistance Publique—Hôpitaux de Paris, Université Paris Cité, Paris, France; Department of Thoracic Surgery and Lung Transplantation, Hôpital Européen Georges-Pompidou, Assistance Publique—Hôpitaux de Paris, Université Paris Cité, Paris, France; Department of Thoracic Surgery and Lung Transplantation, Hôpital Européen Georges-Pompidou, Assistance Publique—Hôpitaux de Paris, Université Paris Cité, Paris, France; Department of Thoracic Surgery and Lung Transplantation, Hôpital Européen Georges-Pompidou, Assistance Publique—Hôpitaux de Paris, Université Paris Cité, Paris, France; Department of Thoracic Surgery and Lung Transplantation, Hôpital Européen Georges-Pompidou, Assistance Publique—Hôpitaux de Paris, Université Paris Cité, Paris, France; Centre de Recherche des Cordeliers, INSERM UMR-S 1138, Sorbonne Université, USPC, Université Paris Cité, Paris, France

**Keywords:** Middle lobe suffering, Lobar malposition, Right upper lobectomy, Lung cancer, Morbidity, Postoperative

## Abstract

Middle lobe (ML) suffering after right upper lobectomy (RUL) is rare but represents a major complication usually due to lobar torsion. We report 3 atypical consecutive cases of ML suffering due to malposition of the 2 remaining right lobes with a 180° tilt. All 3 female patients had surgery for non–small-cell carcinoma including RUL associated with radical hilar and mediastinal lymph node removal. Postoperative chest X-ray abnormalities appeared at days 1–3 respectively. The diagnosis of malposition of the 2 lobes was done on contrast-enhanced chest CT scan at days 7, 7 and 6, respectively. A reoperation for suspected ML torsion was required in all patients. Three repositionings of the 2 lobes and 1 middle lobectomy were performed. The postoperative courses were then uneventful, and the 3 patients were alive at a mean follow-up of 12 months. Before thoracic approach closure after RUL, systematic check of good positioning of the 2 reinflated remaining lobes is indispensable. It may prevent ML suffering secondary to 180° lobar tilt leading to whole pulmonary malposition.

## INTRODUCTION

Lobe torsion first described by Epplen and Jacobson [1] in 1930 corresponds to a twist of the lobe on its bronchovascular pedicle leading to ischaemia, infarction and potential fatal evolution. We report 3 atypical consecutive cases of middle lobe (ML) suffering not corresponding to an ML torsion (MLT) but to a malposition of the 2 remaining right lobes due to a 180° lobar tilt.

## PATIENTS’ DATA

The patients’ data were accessed and managed using Research Electronic Data Capture (REDCap) a secure, web-based software platform [2] (https://redcap.egp.aphp.fr). Informed consent was obtained from all the patients and investigations were conducted according to Declaration of Helsinki principle. The study was carried out according to the French laws on biomedical research.

## RESULTS

### Case no 1

**Figure 1:**
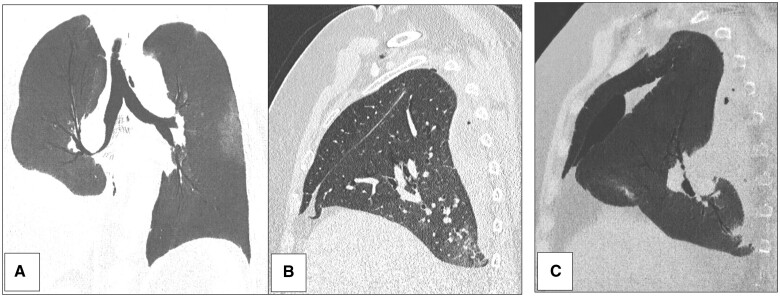
Case no 1: chest CT scan at day 7 with coronal view (**A**) and sagittal views (**B** and **C**). A middle and lower lobe malposition with a 180° lobar tilt were revealed. Middle lobe was in back position. Diaphragmatic face of right lower lobe was close to back part of anterior chest wall and Fowler’s segment was in extreme lower part of the pleural cavity.

A 60-year-old female patient underwent right upper lobectomy (RUL) with Fowler segmentectomy and complete lymph node removal by thoracotomy for pT4N0 adenocarcinoma. The 2 fissures were almost complete. At the end of the procedure, the lung was reinflated and no abnormal tilt of the ML was detected. However, prevention of MLT was done using a resorbable anti-adhesive membrane applied on both lobes. The patient was immediately extubated at the end of surgery and the first 48 postoperative hours were uneventful. Three days after surgery, juxta mediastinal opacity was discovered on chest X-rays. At day 4, moderate biological disorders appeared (high white blood cell count and C-reactive protein level) without clinical abnormalities. Contrast-enhanced chest CT scan was performed at day 7 revealing malposition of middle and lower lobes with ML atelectasis in posterior position (Fig. 1A–C). The Fowler’s segment being completely inverted in a full basal position (Fig. 1B and C). Reoperation was done confirming a 180° rotation counterclockwise of both middle and lower lobes, but the vessels remained permeable. Repositioning of both lobes led to the restauration of parenchyma colouration and good ventilation, allowing to preserve the 2 lobes. The postoperative period was uneventful and at 1 year follow-up the patient is in good condition excepted for neuropathic pains.

### Case no 2

**Figure 2:**
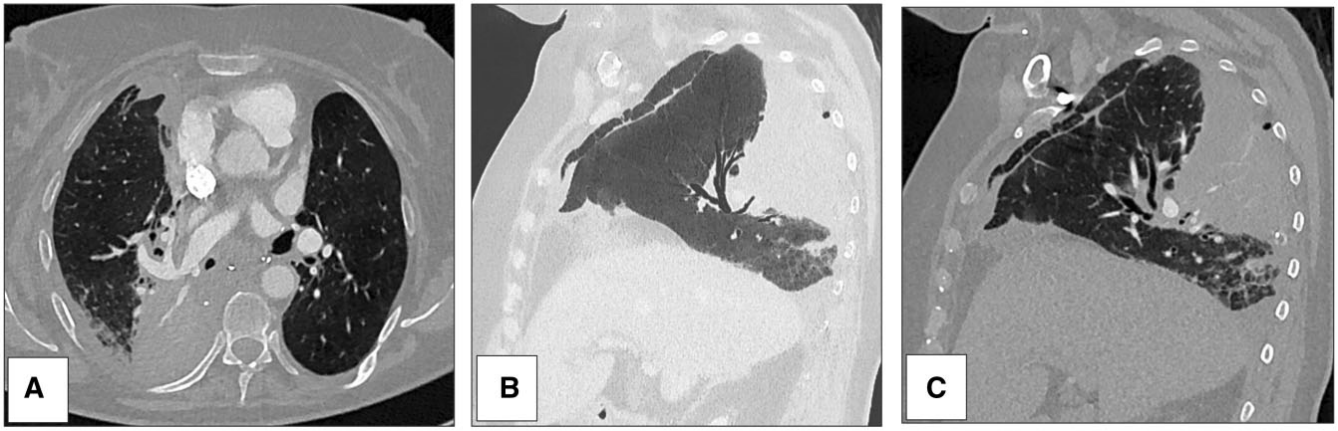
Case no 2: chest CT scan at day 6 with axial view (**A**) and sagittal views (**B** and **C**). A middle and lower lobe malposition with a 180° lobar tilt were revealed. Middle lobe positioned in the back part of pleural cavity presented signs of infarction. Diaphragmatic face of right lower lobe was close to back part of the anterior chest wall and Fowler’s segment was in extreme lower part of the pleural cavity. The diameter of pulmonary artery for right lower lobe was also reduced (**A**).

A 47-year-old female patient underwent RUL and complete lymph node removal through thoracotomy for pT2aN0 adenocarcinoma. The 2 fissures were incomplete. At the end of the surgical procedure, the lung was reinflated and no pneumopexy was done because no tilt of the ML was observed. On the second postoperative day, an ML atelectasis was seen on chest X-ray. Fever and inflammatory biological disorders started the next day. Despite increasing physiotherapy, global condition worsening justified to perform a contrast-enhanced chest CT scan. It revealed a middle and lower lobe malposition with a 180° lobar tilt and ML signs of infarction (Fig. 2A–C). The diaphragmatic face of the right lower lobe was in anterior position close to the anterior chest wall and the Fowler’s segment in basal position (Fig. 2B and C). The bronchovascular pedicle was rotated in an inappropriate direction and the diameter of pulmonary artery for right lower lobe was reduced. At immediate re-exploration, we found a complete 180° rotation of both middle and lower lobes. Repositioning of both boles did not allow to improve the ML infarction. A middle lobectomy was done. The postoperative period was uneventful and at 17 months after surgery the patient is in good condition.

### Case no 3

A 67-year-old female patient underwent extended pneumolysis before RUL and complete lymph node removal for central tumour corresponding to typical carcinoid pT1bN1 through video-assisted thoracoscopy. The 2 fissures were highly incomplete. At the end of the surgical procedure, the lung was reinflated and no prophylactic pneumopexy was performed because no abnormal tilt of the ML was observed. Since the first postoperative day, ML atelectasis had been observed despite intensification of respiratory physiotherapy. Fever and inflammatory biological disorders started from the third postoperative day. Because no improvement occurred after bronchoscopic aspiration of purulent sputum, a contrast-enhanced chest CT scan was performed. A malposition of middle and lower lobes was discovered associated to ML atelectasis. The bronchovascular pedicle was rotated in an inappropriate direction revealing reduced venous circulation from the lower lobe. Reoperation was decided revealing the diaphragmatic surface of the lower lobe facing the posterior part of the thoracic cavity with Fowler’s segment positioned downwards, and an ML atelectasis. Vessels remained permeable with no obvious thrombus inside. Repositioning of the middle and lower lobes was possible with immediate improvement of the parenchyma colouration and good ventilation. The postoperative period was uneventful and the woman is in excellent condition at 11 months follow-up.

## DISCUSSION

The 3 cases we have reported correspond to a suffering of ML due to malposition of the 2 remaining lobes after RUL. This phenomenon is little described in the literature which mainly reports cases of isolated MLT [3]. Whether the mechanism involved is torsion or malposition, surgical re-exploration is required without delay for ML recovery avoiding mortality. The importance of performing a contrast-enhanced chest CT scan early was also reported by Moser and Proto [4]. Despite a diagnosis which was finally quite late in our 3 patients, by CT scan carried out between day 6 and day 7, there was no death. In 2016, Dai *et al.* [3] reported a meta-analysis pooling 91 studies about MLT with an average mortality rate of 8.8%. The main feared risk common to torsion and malposition is the venous occlusion because the middle vein has a small size. This risk is well known by thoracic surgeons performing RUL. The venous occlusion leads to congestion and alveolar haemorrhagic oedema and finally parenchymal infarction. In our cases, a repositioning was first tried for all patients and only 1 lobectomy was done because of lobe infarction. Some investigators may argue against detorsion because of the risks of embolism secondary to emboli released from the occluded pulmonary vein, and also because of the risk of ARDS and possibly multi-organ dysfunction secondary to an acute inflammatory reaction [5]. In their paper, Dai *et al.* [3] reported no significant difference in mortality between the patients who underwent resection and those who underwent detorsion. The choice depends on the operative findings. It is likely that MLT alone without tilting of the lower lobe represents a greater risk of early and permanent occlusion of the ML vein because of the smallness of the pedicle that closes immediately.

Lobe torsion could occur in 3 different circumstances: as a complication of thoracic surgery including lung transplantation, after blunt traumatism and spontaneously.

It is a rare complication often underestimated. Wong and Goldstraw [6] reported that only 30% of thoracic surgeons had seen cases of lobe torsion based on a survey of 117 thoracic surgeons in the UK. The true incidence of lobe torsion after pulmonary resection is difficult to determine. Larsson *et al.* [7] reported 4 cases in 2000 thoracotomies with an incidence of 0.2%. Cable *et al.* [8] reported 7 cases of 7887 patients who underwent pulmonary resection with an incidence of 0.089%.

In our series, the average time between the initial RUL and the diagnosis of lobar mispositioning was 7 days. This delay appears to be too long for an event that occurs immediately before chest closure during initial surgical procedure, but it is consistent with other series reporting MLT [3]. This diagnostic delay is probably due to the heterogeneity of the clinical presentations that seem to be silent at the beginning. On the opposite, other authors have suggested that lobe torsion could present a dramatic picture in the early postoperative period with immediate deterioration like acute respiratory failure. The mechanisms involved in lobar torsion and malposition of the 2 remaining right lobes are probably different. In his study, Felson [9] described 4 predisposing factors as mechanisms of lobar torsion: an airless lobe; a long, free lobe pedicle; an absence of a parenchymal bridge between contiguous lobes; a pneumothorax or pleural effusion; and the transection of the inferior pulmonary ligament.

None of our patients had >2 of these factors. All patients had a transection of the inferior pulmonary ligament to access the inferior lymphatic nodes. One patient had complete fissure and no parenchymal bridge between the middle and lower lobes and underwent pneumopexy. That is, the reason why we considered that the malposition of both ML and lower lobes is due to a poor orientation of the lung by the surgeon at the end of the surgical procedure. In fact, during surgery the lung endures different positions in the chest, resulting in temporary reversal of the various lobes. In the end, the remaining lung is being inflated and cannot spontaneously come back to its correct anatomical position. The presence of a thoracostomy tube under suction, inducing negative pressures, may contribute to this effect especially if it is badly positioned. That is why a good positioning of the ML before thoracic closure is essential for all patients even for those operated by VATS. Eguchi *et al.* [10] who first reported an experience of lobe torsion after VATS resection wondered if the confirmation of good positioning might be less accurate than under direct vision because of less free space in the thorax during reinflation. However, VATS or robotic lobectomy has now emerged for a long time as an increasingly successful approach in the management of this complication and no more lobar torsion was reported. Usually, prophylactic lung fixation can be proposed for patients with multiple predisposing factors as a successful method to prevent lung torsion using different techniques. However, these techniques are useless to prevent bi-lobar malposition. The only prevention is to check the correct positioning of the 2 reinflated lobes at the end of RUL before the chest wall closure.

Systematic prospective analysis of all radiologic data after RUL may have a great interest to better understand and correctly interpret postoperative radiologic abnormalities as early as possible.

## CONCLUSION

Any suspicion of torsion or malposition of the remaining lobes after RUL represents an emergency for the diagnosis and urgent reoperation after carrying out contrast-enhanced CT scan.

**Conflict of interest:** none declared.

**Reviewer information:** Interdisciplinary CardioVascular and Thoracic Surgery thanks Apostolos Nakas, Amr A Arafat and the other anonymous reviewer(s) for their contribution to the peer review process of this article.
